# Differences in Sulfotyrosine Binding amongst CXCR1 and CXCR2 Chemokine Ligands

**DOI:** 10.3390/ijms18091894

**Published:** 2017-09-03

**Authors:** Natasha A. Moussouras, Anthony E. Getschman, Emily R. Lackner, Christopher T. Veldkamp, Michael B. Dwinell, Brian F. Volkman

**Affiliations:** 1 Department of Microbiology and Immunology, Medical College of Wisconsin, Milwaukee, WI 53226, USA; nmoussouras@mcw.edu; 2 Department of Biochemistry, Medical College of Wisconsin, Milwaukee, WI 53226, USA; agetschman@mcw.edu; 3 Department of Chemistry, University of Wisconsin-Whitewater, Whitewater, WI 53190, USA; LacknerER29@uww.edu (E.R.L.); veldkamc@uww.edu (C.T.V.)

**Keywords:** CXCL5, CXCL8, CXCR1, CXCR2, sulfotyrosine, post-translational modification, chemokines, NMR

## Abstract

Tyrosine sulfation, a post-translational modification found on many chemokine receptors, typically increases receptor affinity for the chemokine ligand. A previous bioinformatics analysis suggested that a sulfotyrosine (sY)-binding site on the surface of the chemokine CXCL12 may be conserved throughout the chemokine family. However, the extent to which receptor tyrosine sulfation contributes to chemokine binding has been examined in only a few instances. Computational solvent mapping correctly identified the conserved sulfotyrosine-binding sites on CXCL12 and CCL21 detected by nuclear magnetic resonance (NMR) spectroscopy, demonstrating its utility for hot spot analysis in the chemokine family. In this study, we analyzed five chemokines that bind to CXCR2, a subset of which also bind to CXCR1, to identify hot spots that could participate in receptor binding. A cleft containing the predicted sulfotyrosine-binding pocket was identified as a principal hot spot for ligand binding on the structures of CXCL1, CXCL2, CXCL7, and CXCL8, but not CXCL5. Sulfotyrosine titrations monitored via NMR spectroscopy showed specific binding to CXCL8, but not to CXCL5, which is consistent with the predictions from the computational solvent mapping. The lack of CXCL5–sulfotyrosine interaction and the presence of CXCL8–sulfotyrosine binding suggests a role for receptor post-translational modifications regulating ligand selectivity.

## 1. Introduction

Chemokines comprise a family of approximately 50 small globular proteins that coordinate the migration of immune cells along an increasing chemokine concentration gradient by activating specific G protein-coupled receptors (GPCRs) expressed on the surface of responding cells. The two main classes of chemokines and their receptors, CC and CXC, exhibit varying degrees of promiscuity, with some receptors binding multiple ligands, and certain ligands binding multiple receptors [1,2,3]. Chemokines adopt a highly conserved tertiary fold comprised of a three-stranded antiparallel β-sheet and a C-terminal α-helix stabilized by one or two disulfide bonds. Receptor binding and activation is described by a two-site, two-step model, whereby the N-terminus of the receptor binds the N-loop and chemokine core (site 1), followed by the insertion of the flexible N-terminus of the chemokine into the orthosteric pocket of the GPCR (site 2), leading to receptor activation [4].

**Figure 1:**
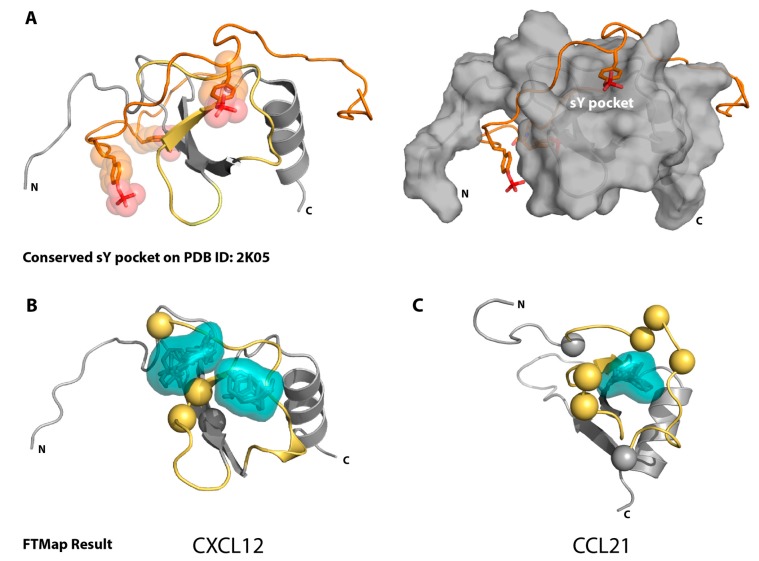
FTMap correctly identifies the NMR-verified sulfotyrosine-binding pocket on CXCL12 and CCL21. (**A**) The solved solution structure of CXCL12 bound to the CXCR4 N-terminal peptide (residues 1-38, sY7, sY12, sY21; Protein Data Bank (PDB) ID: 2K05), left. The CXCR4 N-terminal peptide and surface view of CXCL12 with the sY-binding pocket labeled are shown in the right panel. The tyrosine side chains of the CXCR4 peptide are displayed, the sulfate group is highlighted in red, the canonical sY-binding pocket highlighted in yellow; (**B**) First and fourth top-ranking FTMap clusters (shown in teal) map to the CXCL12 (PDB ID: 2K05) sY-binding pocket (backbone highlighted in yellow) identified by NMR sulfopeptide studies. Spheres reflect specific residues identified by NMR sY titrations [16]; (**C**) Third top-ranking FTMap cluster maps to the CCL21 sY-binding pocket (backbone highlighted in yellow), identified by NMR sY titrations (specific residues highlighted with spheres [30]) (PDB ID: 2L4N).

For some chemokine receptors, tyrosine residues in the N-terminal domain (site 1) are post-translationally modified by tyrosyl protein sulfotransferases [5,6,7], and for the large majority that have been characterized, tyrosine sulfation enhances chemokine–receptor recognition [8,9,10,11,12,13,14]. NMR studies of CXCL12 bound to the N-terminal extracellular domain of its receptor CXCR4 provided the first structural details of sulfotyrosine (sY) recognition by a chemokine [12,13]. Of the three tyrosines in the CXCR4 N-terminal domain (Y7, Y12, and Y21) that are potential sites of sulfation, Y21 is the most important for CXCL12 binding [15]. Y21 makes specific contacts with the N-loop and β3 strand, which may represent a conserved “hot spot” for receptor binding in the chemokine family (Figure 1A) [16]. More recently, the NMR structure of a CCR3-chemokine complex demonstrated that a pair of adjacent sulfotyrosines occupied the same N-loop/β3 cleft of CCL11 [17]. In the case of CCR3, different patterns of sulfation for its two N-terminal tyrosines enhanced the site 1 binding affinity for its ligands CCL11, CCL24, and CCL26 to varying degrees [11]. Thus, tyrosine sulfation can increase the selectivity of a promiscuous receptor by promoting interactions with a subset of its cognate chemokine ligands [11].

A subset of CXC chemokines positive for the ELR (Glu-Leu-Arg) motif are potent neutrophil chemoattractants that activate the CXCR1 and/or CXCR2 receptor [18,19] and play critical roles in inflammatory responses, particularly in response to bacterial infections and autoimmune diseases [18,20,21]. Specifically, CXCL5, which binds both CXCR1 and CXCR2 [22], has been implicated in mediating pain in rheumatoid arthritis and UVB irradiation, and insulin resistance in obesity [23,24,25]. CXCR2, the most promiscuous of the six known CXC chemokine receptors, binds to CXCL1, CXCL2, CXCL3, CXCL5, CXCL6, CXCL7, and CXCL8 [1]. In contrast, CXCR1 predominantly binds CXCL8 and CXCL6, though CXCL5 is reported as a ~10-fold less potent ligand [22,26]. The CXCR2 N-terminal domain contains two tyrosines, neither of which is a likely candidate for sulfation based on local sequence analysis by Sulfinator [27] and Sulfosite [28], while the position-specific scoring matrix (PSSM) developed by Liu et al. [29] gives an intermediate sulfation likelihood score. In contrast, the Sulfosite algorithm predicts that the single tyrosine, Y27, of CXCR1 will be modified with a 92% probability, and similarly, the PSSM also predicts Y27 sulfation with high scoring. While tyrosine sulfation has not been experimentally verified for CXCR1, we speculated that the potencies of CXCL5 and CXCL8 as CXCR1 agonists might correlate with the relative importance of sulfotyrosine recognition for each chemokine ligand.

We have previously validated the modified amino acid sulfotyrosine as a chemical probe in 2D NMR studies [16]. Sulfotyrosine binding to CXCL12 induced chemical shifts in a subset of the residues that were also perturbed upon the binding of CXCR4-derived sulfopeptides [15]. Based on their location in the N-loop/β3 cleft, we concluded that the amino acid probe bound at or near the location of sulfotyrosine 21 in the structure of a CXCR4 sulfopeptide bound to CXCL12 [12]. So far, each chemokine tested (CXCL12, XCL1, CCL5, CX3CL1, and CCL21 [16,30]) bound the sulfotyrosine probe in at least one common pocket, which is consistent with our hypothesis that sulfotyrosine recognition is a conserved feature of the chemokine–receptor site 1 interface (Figure 1). Computational solvent mapping analysis confirmed this hypothesis by clustering organic solvent probe molecules in and around the conserved sulfotyrosine binding sites on the surfaces of CXCL12 [31] and CCL21 [30]. In the present study, computational solvent mapping consistently identified a similar binding pocket on all CXCR2 ligands with available structures, with the exception of CXCL5. Consistent with the computational hot spot analysis, we observed specific binding of sulfotyrosine to CXCL8, but not CXCL5, as monitored by 2D NMR. These findings suggest that receptor recognition by CXCL5 may differ from the other CXCR1/2 chemokine ligands, and that sulfotyrosine may help CXCR1 discriminate between CXCL5 and CXCL8.

## 2. Results

### 2.1. Sulfotyrosine Recognition Sites Correspond to Predicted Chemokine Hot Spots

Previous computational solvent mapping of CXCL12 and CCL21 using FTMap identified sulfotyrosine recognition sites as hot spots for ligand binding [30,31]. The FTMap algorithm surveys the protein surface with 16 small organic molecule probes, then clusters and ranks these probes based on positions with the lowest Boltzmann averaged energies, the highest ranking having the lowest energy [32,33]. We began by using the FTMap server (ftmap.bu.edu) to validate the analysis by testing CXCL12 and CCL21, for which there are both FTMap and sulfotyrosine/sulfopeptide-binding data available (Figure 1). Indeed, the top-ranking FTMap clusters localized to the sulfotyrosine-binding pocket of both of these representative CXC and CC chemokines, which indicated that the lowest energy-binding hot spot was likely to be the sulfotyrosine-binding pocket. 

**Figure 2:**
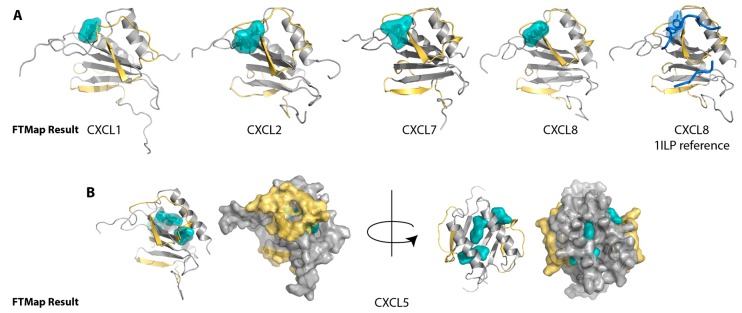
FTMap hot spot identification on CXCR2 chemokine ligands. The canonical sulfotyrosine-binding pocket between the N-loop and β3-strand is highlighted in yellow. The clusters that bind the pocket, each from within the top three ranking clusters, are in teal. (**A**) The clusters find the pockets for CXCL1, CXCL2, CXCL7, and CXCL8. For reference, the structure of CXCL8 bound to the CXCR1 N-terminal peptide (PDB ID: 1ILP) is shown with the peptide in blue and the tyrosine side chain revealed; (**B**) In comparison for CXCL5, the top three-ranking clusters localize to the dimer interface. The surface view is shown to underscore the lack of clusters in the binding pocket.

**Figure A1:**
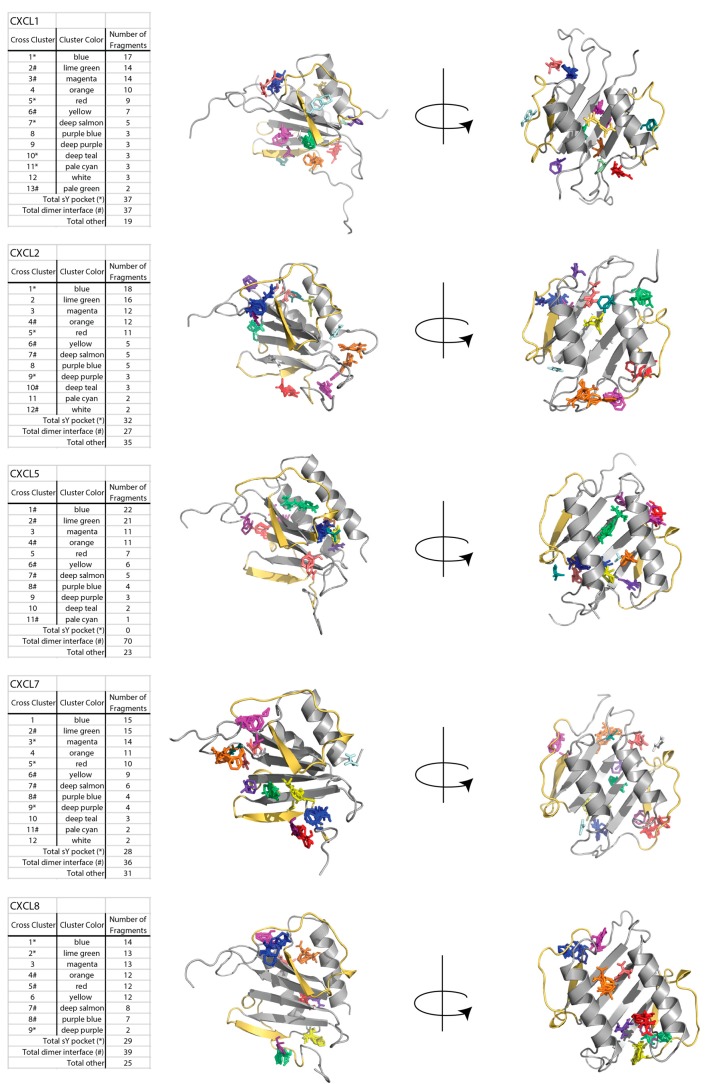
Complete FTMap results. All FTMap clusters are shown on the structures from Figure 2. The higher the number of probes, or fragments, in each cluster, the higher the ranking of that hot spot. Clusters that bind the sulfotyrosine binding pocket are noted with an asterisk (*), and totaled at the bottom of the table. Clusters that bind the dimer interface are noted with a number sign (#), and totaled. The remaining clusters are also totaled at the bottom of the table.

We next analyzed each of the CXCR2 chemokine ligands for which a solved 3D structure was available, including all members of the NMR ensemble where applicable. As shown in Figure 2A, the top-ranking clusters identified by FTMap for CXCL1, CXCL2, CXCL7, and CXCL8 were consistently located between the N-loop and β3-strand near the conserved sulfotyrosine-binding site. While a solved structure of sulfated CXCR1 or CXCR2 (or the sulfopeptide region of the receptor) with any of their ligands has not been solved, the solved NMR structure of CXCL8 with an N-terminal CXCR1 peptide [34] shows the tyrosine near the proposed sulfotyrosine-binding pocket, at the same location of many of the FTMap clusters (Figure 2A). These clusters include those of CXCL1, which lie slightly behind the N-loop, but are still within range that might encounter a CXCR2 peptide. This tyrosine of CXCR1 does not rest in the same position on CXCL8 as the tyrosine 21 of CXCR4 (PDB ID: 2K04) [12], which may account for the different positioning of the clusters relative to those in the CXCL12 analysis. For CXCL5 (Figure 2B), however, only the last, or second to last-ranking cluster identified the pocket, which suggested that for CXCL5, it is a less favorable binding pocket. Furthermore, clusters only found the pocket for four of the 20 CXCL5 NMR conformers, as compared to a majority of conformers in the CXCL1, CXCL2, and CXCL8 NMR ensembles (Figure A1).

### 2.2. NMR Titration Studies with Sulfotyrosine

**Figure 3:**
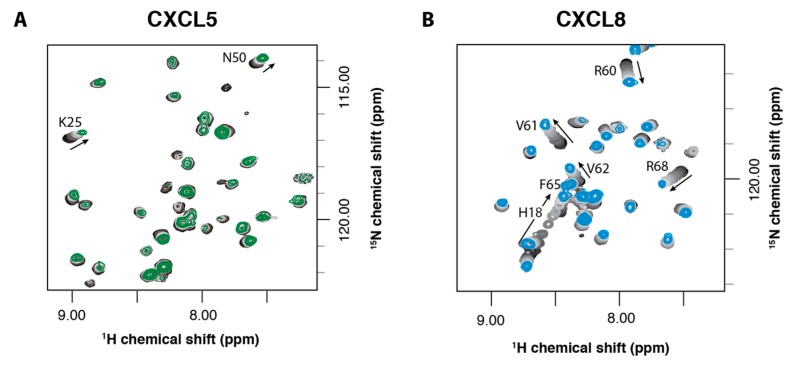
NMR titrations of CXCL5 and CXCL8 with sulfotyrosine (sY). Overlays of the ^1^H-^15^N HSQC spectra of CXCL5 (**A**) or CXCL8 (**B**) in the presence of 0 mM sY (black), 1, 5, 10, 20, 30, 40, 60, 80 and 100 mM sY (green or blue, respectively). A region of the HSQC spectra is shown to highlight that while there are some shift perturbations in (**A**); there are widespread changes in the CXCL8 **(B)** spectra.

**Figure A2:**
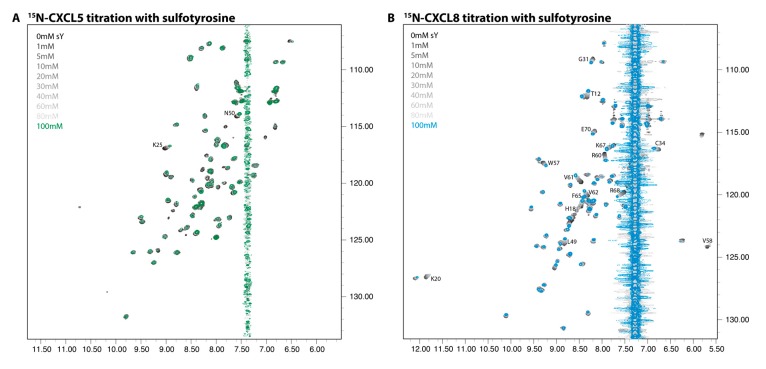
Complete HSQC overlays of titrations of sulfotyrosine (sY) into CXCL5 (**A**) or CXCL8 (**B**) from 0 (black) to 100 mM (green or blue, respectively). The streak around 7.4 ppm is due to increasing concentrations of sY, which are indicated in the figure. Residues with CSPs >0.3 ppm are labeled (see also Figure 4A,B).

To further explore these results, we performed NMR titration experiments to similarly probe chemokine receptor-binding pockets with another small molecule, sulfotyrosine. We have previously shown that sulfotyrosine can be used as a surrogate for sulfated receptor peptides and is sufficient to identify the chemokine receptor sulfotyrosine-binding pocket on the chemokine ligand [16]. Using ^1^H-^15^N HSQC NMR spectroscopy, we monitored the spectra of CXCL5 upon titration of increasing amounts of sulfotyrosine, from 0 to 100 mM. The amino acids involved in binding were expected to show the greatest chemical shift perturbations. However, when performing these titrations with CXCL5, there were few residues that had significant chemical shift perturbations (Figure 3A, Figure A2–A). For comparison, we performed a similar titration for CXCL8 and, as predicted from our prior studies of CXCL12, there were widespread changes in the HSQC spectra throughout the course of the titration (Figure 3B, Figure A2–B). NMR HSQC experiments are superbly sensitive to changes in protein structure. For these experiments, as the only change throughout the titration was the addition of sulfotyrosine, the changes in the spectra are indicative of sulfotyrosine binding.

**Figure 4:**
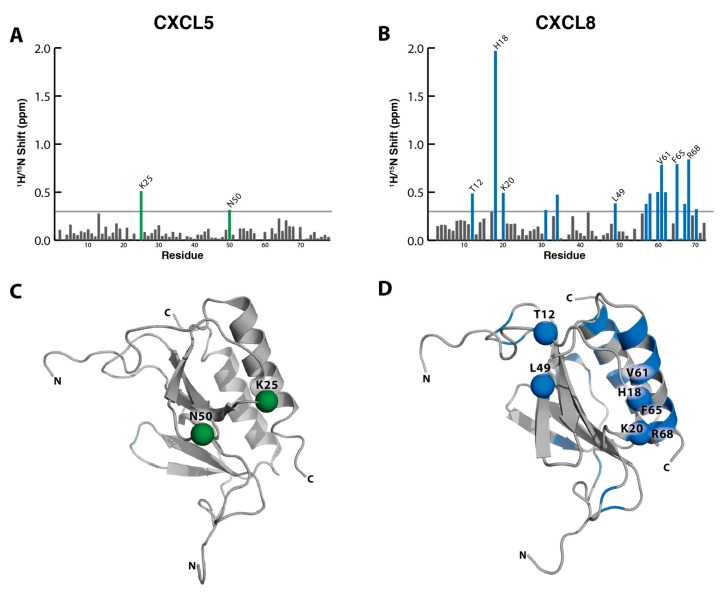
Total chemical shift perturbations (CSP) from 0 to 100 mM sulfotyrosine (sY) plotted for each amino acid. (**A**) CXCL5 CSP plot, residues in green reflecting CSP >0.3 ppm; (**B**) CXCL8 CSP plot, residues in blue reflecting CSP >0.3 ppm; (**C**) CXCL5 structure (PDB ID: 2MGS) with K25 and N50, residues with the largest chemical shift perturbations (>0.3 ppm) within the sY-binding pocket are highlighted with green spheres; (**D**) CXCL8 (PDB ID: 2IL8) structure highlighting residues with the highest chemical shift perturbations (>0.3 ppm) in blue. Residues of the sY-binding pockets T12, H18, K20, and L49, are shown as spheres.

As a measure of sulfotyrosine–chemokine interactions, total (^1^H and ^15^N) chemical shift perturbations can be quantified and magnitudes plotted as a function of residue number. For the CXCL5 titration, there were very minor changes in the spectra throughout the course of the titration, resulting in small chemical shift perturbations mostly within the level of noise (Figure 4A). When we mapped the amino acid residues K25 and N50 onto the structure of CXCL5 (PDB ID: 2MGS), interestingly, they do map to the edge of the sulfotyrosine-binding pocket (Figure 4C). The few, small chemical shift perturbations observed are likely due to the non-specific coordination of the negatively charged free sulfotyrosine and positively charged amino side chain of K25. 

In contrast, the addition of sulfotyrosine to CXCL8 produced chemical shift perturbations indicative of specific sulfotyrosine binding [12,13]. There were regions of the N-loop and β3 strand (T12, H18, K20, and L49) encircling the canonical sulfotyrosine-binding pocket that produced significant (>0.3 ppm) chemical shift perturbations. As opposed to CXCL5, these were far above the level of noise. They closely overlap with residues that bind a CXCR1 N-terminal peptide as observed by Joseph et al., which fits a model of receptor–sulfation binding at the sulfotyrosine-binding pocket [35]. There were additional significant chemical shift perturbations in the C-terminal helix cluster (W57, V58, R60, V61, V62, F65, K67, R68, E70) adjacent to the N-loop, which may be the result of sulfotyrosine binding to the N-loop/β3 cleft or could denote a second binding site (Figure 4B,D).

### 2.3. Binding Affinity to Sulfotyrosine

**Figure 5:**
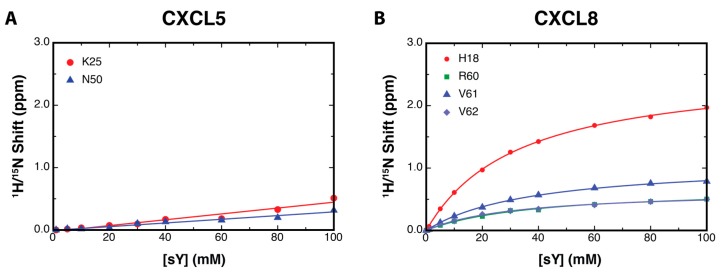
Sulfotyrosine–chemokine binding affinities. *K*_d_ plots of the CXCL5 (**A**) and CXCL8 (**B**) amino acids with the largest chemical shift perturbations indicating no saturable binding of sulfotyrosine (sY) to CXCL5, but did indicate saturable binding to CXCL8. The titration of sY into CXCL8 produced a *K*_d_ of 35.2 ± 1.95 mM.

**Figure A3:**
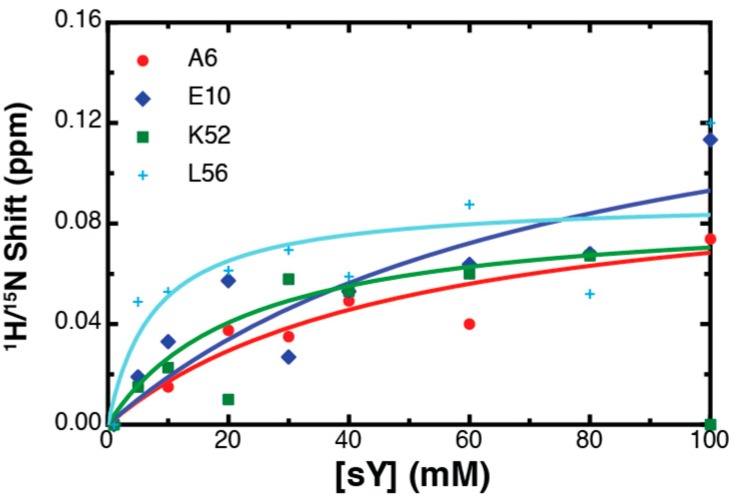
*K*_d_ plot showing curves with the lowest error from the titration of sY into CXCL5. These data further suggest CXCL5 had no saturable, specific binding.

For an NMR titration that exhibits fast exchange kinetics and reaches a point of saturation (where the addition of a ligand produces little or no spectral changes), the chemical shift perturbations at intermediate titration points reflect the fractional occupancy of a binding site, and can be used to generate a binding isotherm. Sulfotyrosine-dependent chemical shift perturbations were analyzed by nonlinear fitting to estimate the dissociation constant, *K*_d_ [30]. For CXCL5, K25 and N50 had the greatest chemical shift perturbations throughout the titration. However, using a standard ligand-depletion, saturable-binding model, the data produced a linear curve, indicating non-specific interactions between sulfotyrosine and CXCL5 (Figure 5A). While non-specific interactions may occur due to the relatively small size of sulfotyrosine and lead to observable chemical shift perturbations, even those residues that exhibited smaller perturbations did not produce a saturable binding curve (Figure A3), suggesting that CXCL5 does not bind sulfotyrosine in a specific manner. In comparison, select CXCL8 residues generated large chemical shift perturbations, resulting in saturable binding curves (Figure 5B). When calculated binding affinities were averaged, they produced a binding *K*_d_ of 35.2 ± 1.95 mM, which is comparable to other sulfotyrosine titrations [30]. 

## 3. Discussion

The mechanism by which promiscuous chemokine receptors selectively bind individual ligands remains poorly understood. A combination of factors including chemokine concentration, glycosaminoglycan (GAG) interactions, oligomerization state, and other cellular, contextual, or kinetic variables may fine-tune the propensities for chemokine–receptor interactions encoded by the amino acid sequence of each chemokine ligand. For example, CXCL5 expression was slower and more sustained compared with those of CXCL1 or CXCL8 in bacterial-infected epithelium [36,37]. This difference reveals not only the importance of chemokine expression, but also receptor selectivity, as there are often multiple ligands present simultaneously [38,39,40]. Post-translational modifications to the extracellular domains of the receptor are an emerging biologic paradigm that influences ligand–receptor binding kinetics, selectivity, specificity, and signaling [10,12,41,42]. The goal of the present study was to examine the potential role of N-terminal tyrosine sulfation of CXCR1 and CXCR2 in binding CXCL5 and CXCL8.

Tyrosine sulfation is an established receptor modification that we as well as others have shown to increase the affinity of a chemokine for the N-terminal domain of its cognate receptor. We used an unbiased computational solvent mapping approach to identifying hot spots on chemokine surfaces that consistently matched a known sulfotyrosine-binding site [13,16]. This same hot spot was predicted for all CXCR2 ligands, except for CXCL5. Previous studies have not only uncovered a role for chemokine receptor tyrosine sulfation, but also validated the use of sulfotyrosine as a useful molecular probe for the discovery of receptor-binding sites [16]. In striking contrast to CXCL8 and all the other chemokines tested to date [13,16,30], CXCL5 exhibited no signs of specific sulfotyrosine binding in NMR titrations. Sulfotyrosine-induced perturbations correspond closely with CXCL8 N-loop/β3 residues that shifted in a titration with a CXCR1 N-terminal peptide [35], as well as residues in the C-terminal helix that bind heparin oligosaccharides [43]. Often, there are regions of overlap between the binding of chemokine ligands with a receptor N-terminal peptide and GAGs, including for CXCL8 [35,43,44,45,46]. The pattern of shifts in both areas of CXCL8 suggests that sulfotyrosine may mimic both the modified receptor and sulfate-rich GAGs. 

Sulfosite and the PSSM sulfation prediction sources predict CXCR1’s tyrosine sulfation [28,29], and the PSSM predicts CXCR2’s tyrosine sulfation [29]. Based on these bioinformatics analysis tools, it is likely that CXCR1 is tyrosine-sulfated, and less likely that CXCR2 is tyrosine-sulfated. However, both may be sulfated in vivo [5]. CXCL8 has a ~1–4 nM affinity at both CXCR1 and CXCR2 and is the most potent ligand at CXCR1, while CXCL5 has an affinity of ~40 and ~11 nM, respectively [22,26,47]. The data from the CXCL8 sulfotyrosine titration suggests that this may be due to the sulfation increasing its affinity, as shown previously for sulfated N-terminal receptor peptides and the following receptor/chemokine pairs: CCR2/CCL2 [10] and CCL7 [8], CCR3/CCL11 [9,11], CCL24 [11] and CCL26 [9,11], CCR5/CCL5 [14], and CXCR4/CXCL12 [12,13,15]. Thus, the binding events and affinities between the chemokines and the post-translationally modified receptors may present a nuanced form of regulation that is unique to different physiologic states and each particular chemokine. 

Our FTMap analysis revealed a binding hot spot along the N-loop and β3-strand for the CXCR2-binding chemokine ligands CXCL1, CXCL2, CXCL7, and CXCL8 that corresponds to the canonical sulfotyrosine-binding pocket. This pocket identified by FTMap is compatible with a recent model of the CXCR2 N-terminal peptide bound to CXCL7, which shows the N-terminal peptide docked around the N-loop and over the β3-strand [45]. Based on this docking pose of CXCR2, its two tyrosines would face the opposite side of the chemokine around the α-helix and dimer interface. Interestingly, when the CXCR1 sequence is substituted in this model, the predicted sulfotyrosine occupies the canonical sulfotyrosine-binding site. Specifically, an alignment of CXCR1 and CXCR2 reveals that A31 of CXCR2 (A36 using the UniProtKB numbering system (entry: P25025)) corresponds to Y27 of CXCR1, which is the tyrosine predicted to be sulfated. In this model by Brown et al., A31 of CXCR2 rests in the canonical sulfotyrosine-binding pocket of CXCL7. Thus, we speculate that Y27 of CXCR1 interacts with the canonical sulfotyrosine-binding pocket of its ligands, and its sulfation increases its affinity for certain chemokines. While CXCR2 may or may not be sulfated, the N-loop/β3 cleft is predicted by FTMap as a hot spot in the site 1 interface. Taken together with the model of the CXCR2-CXCL7 complex by Brown et al., these results provide a plausible structural explanation for how tyrosine sulfation of CXCR1, but not CXCR2, might be compatible with the use of a conserved binding pocket by both receptors on promiscuous chemokine ligands. 

The lack of FTMap identification of the binding pocket of CXCL5 and the differences in sulfotyrosine binding between CXCL5 and CXCL8 are due to more than differences in receptor binding as CXCL5 does bind both CXCR1 and CXCR2 (~40 vs ~11 nM, respectively) [22,26]. Sepuru et al. recognize that CXCL5 is more electrostatically neutral than any other CXCR2-activating chemokine [48]. As sulfotyrosine is a negatively charged molecule, it is likely to bind positively charged basic residues. In the region of the N-loop and β3-strand, CXCL8 has basic residues K11, K15, H18, K20, and R47, most of which are perturbed by sulfotyrosine binding or adjacent to a perturbed residue. Basic residues in this region of CXCL5 include H23, K25, and K52, which align with H18, K20, and R47 of CXCL8. Thus K11 and K15 of CXCL8 are residues that may confer sulfotyrosine specificity. Interestingly, CXCL6 is the only other ELR+ chemokine with a basic residue (R20) that corresponds to K15 of CXCL8; this position is invariably a glycine in the other CXCR2 ligands [48]. When Wolf et al. mutated R20 of CXCL6 to G, they found a loss of signaling at CXCR1, with no change in effect at CXCR2 [22], and furthermore, when Jiang et al. mutated K15 of a CXCL8 peptide to A, they found a greater than six-fold decrease in binding affinity to a CXCR1 peptide, as measured by surface plasmon resonance (SPR) [49]. These results are consistent with a specific role for R20 of CXCL6 and K15 of CXCL8 in sulfotyrosine recognition, and their higher potency as CXCR1 agonists relative to the other ELR+ ligands. The more electrostatically neutral, and less basic N-loop/β3 pocket of CXCL5 may account for the lack of sulfotyrosine binding, lack of probe binding between the N-loop and the β3-strand in FTMap, and the overall weaker potency at CXCR1 and CXCR2, more so than any other CXCR2-activating chemokine [26]. 

These results highlight the complexity of post-translational modifications as regulators of chemokine signaling. CXCL5 may employ a different combination of site 1 interactions with its receptors than the other chemokine ligands for CXCR1 and CXCR2. We had previously shown that there are differences in sulfated tyrosine affinities amongst multiple tyrosines on the same *receptor* N-terminus [15]. Our results suggest that, for a particular chemokine *ligand*, the sulfation of a tyrosine on its cognate receptor may not play an important role, and supports a novel paradigm in which sulfotyrosine may not universally increase the affinity of the chemokine receptor for its cognate ligand. For promiscuous receptors, this may be a mechanism to distinguish binding between different ligands. There have long been generalizations about chemokine receptor binding and activation; however, as the intricacies of the system become more apparent, the redundancies fade in favor of subtle differences between chemokine ligands. The absence of a sulfotyrosine-binding site distinguishes CXCL5 from the other chemokines that have been examined and may confer unique functional attributes among the ELR+ subfamily. 

## 4. Materials and Methods

### 4.1. FTMap 

The available structures for CXCR1 and CXCR2 chemokine ligands (CXCL1-PDBID: 1MSH, CXCL2-PDB ID: 1QNK, CXCL5-PDB ID: 2MGS, CXCL7-PDB ID: 1NAP, CXCL8-PDB ID: 2IL8, 5D14) as well as CCL21 (PDB ID: 2L4N, 5EKI) and CXCL12 monomer and dimer with CXCR4 sulfopeptide (PDB IDs: 2KEC and 2K05 respectively) as a reference for chemokines that bind sulfotyrosine were downloaded from the RCSB Protein Data Bank (www.pdb.org) [50]. Using PyMOL (Schrodinger, LLC, New York, NY, Version 1.7) [51], the NMR solution structures (PDB IDs: 1MSH, 1QNK, 2MGS, 2IL8) were separated into PDB files of individual states. These and the crystal structures (PDB IDs: 1NAP, 5D14, 5EKI) were submitted to FTMap computational solvent mapping web server (ftmap.bu.edu) to identify potential binding pockets by small molecule sampling [32]. The results were downloaded and analyzed via PyMOL [51]. 

### 4.2. Protein Expression and Purification

Uniformly labeled ^15^N-CXCL5 was expressed and purified as previously described [52]. ^15^N-CXCL8 was supplied by Protein Foundry, LLC (Milwaukee, WI, USA). 

### 4.3. NMR Spectroscopy

Concentrated stock solutions of ^15^N-CXCL5 or ^15^N-CXCL8 in H_2_O were diluted to 250 µM in a solution containing 50 mM deuterated acetic acid (pH 5.0 for CXCL5, pH 5.2 for CXCL8), 10% (*v*/*v*) D_2_O, 0.02% (*w*/*v*) NaN_3_. All data were collected on a Bruker Avance 600 MHz spectrometer equipped with a ^1^H/^15^N/^13^C cryoprobe. ^1^H-^15^N Heteronuclear Single Quantum Coherence experiments were used to monitor a CXCL5 or CXCL8 sample titrated with 0, 1, 5, 10, 20, 30, 40, 60, 80, and 100 mM sulfotyrosine dissolved in the same buffer as above. Spectra were processed using NMRPipe [53]. Using chemical shift assignments from the solved structures [48,54], peaks were tracked using CARA [55]. Total ^1^H-^5^N chemical shift perturbations were computed as [(5Δδ_NH_)^2^ + (Δδ_N_)^2^]^1/2^, where Δδ_NH_ and Δδ_N_ were the total changes in backbone amide ^1^H and ^15^N chemical shifts in ppm, respectively, from 0 to 100 mM sulfotyrosine. Concentration-dependent chemical shift perturbations for CXCL8 residues H18, R60, V61, and V62 upon titration with sulfotyrosine were fit to the following equation, which accounts for ligand depletion:

$$\Delta \delta =\Delta \delta_{\max}\times \frac{\left(K_{d}+\left[\mathrm{CXCL}8\right]+x\right)-\sqrt{{\left(K_{d}+\left[\mathrm{CXCL}8\right]+x\right)}^{2}-4\left[\mathrm{CXCL}8\right]x}}{2\left[\mathrm{CXCL}8\right]}$$

where Δδ is the chemical shift perturbation, Δδ_max_ is the maximum chemical shift perturbation at 100% bound CXCL8, *K*_d_ is the CXCL8 sY dissociation constant, and *x* is the sY concentration. There were no changes in pH for the CXCL8 titration, thus, these changes in chemical shifts were due solely to the addition of sulfotyrosine. Using pro Fit 6.2 and the above equation, the *K*_d_ values and their respective errors were calculated and averaged to produce the reported affinity and error. Amino acids with the highest chemical shift perturbations were mapped onto the structure of CXCL8 using PyMOL. The same process was attempted for CXCL5; however, the chemical shift perturbation did not fit with this equation, but rather with a linear regression model. 

## Abbreviations

CXCL	CXC ligand
CXCR	CXC receptor
CCL	CC ligand
GPCR	G-protein Coupled Receptor
sY	Sulfotyrosine
NMR	Nuclear Magnetic Resonance
HSQC	Heteronuclear Single Quantum Coherence
CSP	Chemical Shift Perturbation
ELR+	Glutamate-leucine-arginine positive
GAG	Glycosaminoglycan
SPR	Surface Plasmon Resonance
